# Economic burden and coping mechanisms by tuberculosis treatment supporters: a mixed method approach from Bono Region, Ghana

**DOI:** 10.1186/s12913-024-10611-1

**Published:** 2024-01-30

**Authors:** Robert Bagngmen Bio, Patricia Akweongo, Augustina Koduah, Augustine Adomah-Afari

**Affiliations:** 1 School of Public Health, University of Ghana, Legon, Accra, Ghana; 2 School of Pharmacy, University of Ghana, Legon, Accra, Ghana

**Keywords:** Bono Region, Coping mechanism, Economic burden, Ghana, Treatment supporters

## Abstract

**Background:**

The Directly Observed Therapy Short Course (DOTS) strategy recommended by World Health Organization for tuberculosis control requires multiple clinic visits which may place economic burden on treatment supporters especially those with low socio-economic status. The End tuberculosis goal targeted eliminating all tuberculosis associated costs. However, the economic burden and coping mechanisms by treatment supporters is unknown in Ghana.

**Objectives:**

The study determined the economic burden and coping mechanism by treatment supporters in Bono Region of Ghana.

**Methods:**

Cross-sectional study using mixed method approach for data collection. For the quantitative data, a validated questionnaire was administered to 385 treatment supporters. Sixty in-depth interviews with treatment supporters to elicit information about their coping mechanisms using a semi-structured interview guide for the qualitative data. Descriptive statistics, costs estimation, thematic analysis and bivariate techniques were used for the data analysis.

**Results:**

Averagely, each treatment supporter spent GHS 112.4 (US$21.1) on treatment support activities per month which is about 19% of their monthly income. Borrowing of money, sale of assets, used up saving were the major coping mechanisms used by treatment supporters. Highest level of education, household size, marital status and income level significantly influence both the direct and indirect costs associated with tuberculosis treatment support. The significant levels were set at 95% confidence interval and *p* < 0.05.

**Conclusion:**

We concludes that the estimated cost and coping mechanisms associated with assisting tuberculosis patients with treatment is significant to the tuberculosis treatment supporters. If not mitigated these costs have the tendency to worsen the socio-economic status and future welfare of tuberculosis treatment supporters.

**Supplementary Information:**

The online version contains supplementary material available at 10.1186/s12913-024-10611-1.

## Introduction

The tuberculosis (TB) epidemic is generalized affecting every region, district and community in Ghana. While there has been a general decline in both incidence and deaths, dropping from 152 cases per 100,000 in 2017 to 136 cases per 100,000 people and mortality from 52 per 100,000 in 2016 to 36 deaths per 100,000 in 2021, regional disparities exist in terms of treatment outcomes [1, 2].

Furthermore, Ghana detected 30% of all cases in 2021 which is less than the Africa regional average of 47% WHO target of 70% [3, 4]. This implies 70% of new cases are missed. Also, the rise in multi-drug-resistant TB in Ghana reflects issues with treatment adherence. Ghana recorded an increase in multi-drug resistant cases, from 77 cases in 2016 to 1,200 in 2021 [2]. Evidence suggests that lack of treatment adherence gives rise to drug resistant tuberculosis [5].

Besides the severe health implications, TB also has a considerable economic impact, leading to poverty worldwide [6]. The financial burden of TB can be overwhelming for affected countries' economies. For example, in Africa, TB-related deaths were estimated to cause a Gross Domestic Product loss of 50.4 billion USD, which is approximately 1.37% of the combined GDP of 47 WHO African Region countries including Ghana [7].

Annually, TB results in a loss of around US$12 billion in global income, with countries having a high prevalence of TB losing about 4–7% of their gross domestic product (GDP) [8]. This puts a significant financial and social strain on those who suffer from the disease, their families, and communities [9]. As a result, the most significant burden of TB falls on young, productive adults who are often unable to work [10, 11]. Furthermore, the responsibility of caring for sick individuals falls on other family members and friends, which ultimately decreases their productivity [11]. Additionally, children are also affected as they have to drop out of school to help support their families, as reported by Arnold and colleagues [11]. Evidence suggests that annually, TB care results in a loss of three to four months of work time and 20 to 30% of household income [12].

In Ghana, the Directly Observed Treatment Strategy (DOTS) has been implemented to aid TB patients. This method involves the use of treatment supporters to ensure proper adherence to the full course of drug therapy [5]. The main goal is to enhance patient outcomes and prevent the development of drug resistance tuberculosis. To implement DOTS, TB patients and their treatment supporters have to make multiple visits to health facilities for at least six months [5]. Treatment supporters are individuals who volunteer to observe TB patients taking medication on a daily basis and encourage them to complete their treatment as scheduled [5]. 

Based on available evidence, it appears that the DOTS strategy has financial implications for the patient and individuals assisting the patient with treatment. For instance, a study conducted in Tanzania found that while tuberculosis patients were satisfied with the DOTS services they received and treatment supporters were willing to supervise another TB patient, they also noted that repeated visits to the health facility placed a financial burden on them [12]. Similar findings were reported in Ethiopia, where tuberculosis patients and their treatment supporters incurred direct costs of $123.0 and $54.26 respectively, and experienced a reduction in productivity time of 10% [13]. A study in Uganda also reported that treatment supporters incurred significant direct and indirect costs associated with supporting TB patients [14].

Although there have been economic burden studies estimating the cost of tuberculosis in developed and developing countries, there are limited published studies that quantify the economic burden of tuberculosis to treatment supporters globally. The emphasis in these studies has generally been on the health system burden and the tuberculosis patient, rather than the cost incurred by individuals providing support to the patient.

Understanding the economic burden and coping mechanisms of treatment supporters in diverse settings is crucial for policymakers interested in TB control. This study aims to shed light on the costs involved in assisting tuberculosis patients with treatment, with a specific focus on Ghana.

To the best of our knowledge, no study has investigated the economic burden and coping mechanisms of TB treatment supporters in Ghana. Therefore, this study seeks to estimate the economic burden and coping mechanisms of TB treatment supporters in the Bono Region of Ghana, with the hope of informing policy.

## Materials and methods

### Study design

Our study used a concurrent mixed-method approach for data collection, consisting of both quantitative and qualitative data. To gather quantitative data, we administered a validated questionnaire to 385 treatment supporters. Additionally, we conducted 60 in-depth interviews with treatment supporters using a semi-structured interview guide to elicit information about their coping mechanisms, which provided qualitative data. Mixed-method approach was chosen based on the objective of our study. The quantitative data allowed us to estimate the costs of treatment support, while the qualitative data enabled us to explore the coping mechanisms used by treatment supporters to reduce the financial burden associated with providing TB treatment support.

### Study setting

This research was carried out in the Bono Region, covering six locations—three districts and three municipalities. These areas are Berekum Municipality, Dormaa Municipality, Sunyani Municipality, Jaman North District, Jaman South District, and Tain District. The latter three districts are rural compared to the urbanized Berekum Municipality, Dormaa Municipality, and Sunyani Municipality. The selection of these districts/municipalities was based on the high prevalence of tuberculosis cases.

Furthermore, the background analysis of the study districts revealed similar economic and demographic factors, allowing for inferences to be made that cover the entire region. The region is majorly driven by farming as the primary economic activity.

In terms of healthcare, the region follows a decentralized structure, with healthcare delivery ranging from regional to community level. The Bono region comprises 11 administrative districts and has a total of 212 health facilities. This includes 1 regional hospital (located in the regional capital of Sunyani), 9 district hospitals, 82 health centers, 56 clinics, 35 private maternity homes, and 112 functional Community-based Health Planning and Services (CHPS) with several other demarcated CHPS spread across the region. Only two out of the 11 districts lack district hospitals—Banda and Sunyani West districts. These health facilities provide both preventive and curative healthcare services to the population within their catchment areas.

In order to standardize the diagnosis and treatment of tuberculosis (TB) throughout the country, a national guideline has been adopted. This guideline specifies TB diagnosis should be done using GeneXpert technique and treatment regimens based on the Directly Observed Treatment Strategy(DOTS) [15]. The DOTS strategy recommends that patients complete their six-month course of treatment under direct observation by a trained TB treatment supporter overseen by health workers [5, 16]. This helps ensure adherence to the full course of drug therapy, improving patient outcomes and preventing drug resistance. During the six-month treatment period, patients and their supporters visit the health facility every two weeks during the two-month intensive phase of treatment to pick up medications, and monthly during the four-month continuation phase for medications and re-examination [17].The national tuberculosis program coordinates all tuberculosis control-related activities in Ghana, including all tuberculosis control activities in the Bono Region.

### Study population

This study focused on adult TB treatment supporters who are 18 years or older and live in the Bono Region of Ghana. Treatment Supporters are individuals who voluntarily observe TB patients taking their medication daily and encourage them to complete their treatment as schedule. 

To be included in the study, participants had to be at least 18 years old and have observed TB patient treatment for a minimum of two months. Anyone who had not provided treatment support for at least two months was excluded from the study.

### Sampling and data collection

For our study, we purposefully selected six hospitals in the Bono Region that had the highest number of TB cases. These districts are likely experiencing high burden of TB which is of public health concern. We conducted research over a three-month period, specifically from July to September 2019. We identified treatment supporters from TB registers at each hospital and contacted them via phone before their scheduled drug ration date. During a visit to pick up their TB drugs at the hospital, we asked for their informed consent. Respondents who agreed to participate in the study were given a validated questionnaire adapted from the WHO [18]. We obtained the necessary sample of 385 respondents. The questionnaire included questions about the direct and indirect costs associated with TB treatment, as well as socio-demographic characteristics such as age, gender, marital status, income level, household size, occupation, level of education, district of residence, and other relevant factors. We administered the questionnaires after the respondents had completed their routine treatment schedule, for their convenience, as they were about to exit the hospital.

We interviewed a total of 60 respondents who did not take part in the quantitative survey were used for the qualitative data, with 10 respondents from each of the six health facilities, using a purposive selection strategy. This allowed us to gather diverse perspectives on coping mechanisms from different locations within the study. In-depth interviews were used to explore open-ended topics on coping mechanisms, using a semi-structured interview guide that provided some structure while allowing for flexibility in the conversation. The interview guide used for this study was developed after extensive review of related literature (attached as a Supplementary file).

The interview guide covered various coping mechanisms, such as selling properties, using saved money for other purposes, borrowing money from support networks, and stopping savings. These topics are relevant to understanding how respondents manage financial challenges related to TB treatment. All interviews were conducted in a hospital setting, ensuring the convenience and comfort of respondents as they visit to pick up TB drugs. The interviews lasted between 30 to 45 min, which is typical for in-depth interviews.

Privacy was maintained during the interviews, interviewing respondents separately and privately as possible, which is crucial for obtaining honest and confidential responses from respondents. Initially, all interview questions were prepared in English, but were translated and explained in the local languages by trained research assistants. This helps ensure that respondents fully understood the questions and could respond in their preferred language.

We obtained consent from each respondent before recording the interviews. The interviews were audio recorded to accurately capture the respondents' responses and nuances in their answers. Field notes were also taken during the interviews to provide additional context and insights. These notes help in the analysis process by providing context and non-verbal cues that may not be captured in audio recordings.

### Sample size determination

To determine the minimum number of treatment supporters required for this study, the formula for proportions used by previous researchers was adopted. The formula is: *n* = z2 p (1-p) / e2. Here, n represents the minimum sample size needed, z is the z statistic for a confidence level of 95% (which equals 1.96), p is the proportion of TB patients with treatment supporters reported in Ghana is 35% [19], and e is the margin of error (which is 5% or 0.05).

Plugging in the values, we get: 3.84 * 0.35 * (1—0.35) / 0.0025, which equals 350. To account for a non-response rate of 10%, 35 was added to the 350 to arrive at a total sample size of 385 respondents required for this study.

Assuming that each tuberculosis patient had one (1) treatment supporter, a sample size proportion to the number of tuberculosis patients in each district was assigned using the formula, $\frac{\mathrm{number\ of\ treatment\ supporters\ in\ each\ district}}{\mathrm{total\ number\ of\ treatment\ supporters\ in\ the\ six\ districts}}\times 385$  

Table 1 presents details of sample size allocated proportion to the number of tuberculosis patients in each district.

**Table 1:** Number of tuberculosis patients on treatment as of 2018 and sample size allocated

No	District	No. of TB Cases	Sample Size	**%**
1	Berekum Municipality	112	68	18
2	Dormaa Municipality	89	54	14
3	Jaman North	78	48	12
4	Jaman South	62	38	10
5	Sunyani Municipality	170	104	27
6	Tain	120	73	19
	TOTAL	631	**385**	**100**

### Description of costs variables

Detailed description of the costs variables is given in Table 2.

**Table 2:** Description of costs variables

Cost Type	Cost Categories	Description
**Direct Cost**	Feeding	Direct out-of-pocket payments for food and water while waiting at the health facility to waiting to pick up drugs
	Travel/transportation	Cost associated with travel to and from the health facility for TB medications
	Accommodation	Direct out-of-pocket payments for lodging( in case treatment supporters did not pick up drug early and have to sleep over the night)
	Other: communication	Direct out-of-pocket payments on mobile credit/phone calls(made to TB patient or health care provider at DOTS center)
**Indirect Cost** (**productivity losses associated with providing TB treatment support)**	Time spent on travel/transportation	Productive time spent on travel to and from health facility for TB medications
	Time spent with TB patient	Productive time spent with TB patient supervising/serving TB medications
	Waiting Time	Productive time spent at health facility waiting for TB medications

(adapted from a hand book on tuberculosis patients cost surveys [15])

### Data analysis

#### Quantitative analysis

To analyze the quantitative data, we used descriptive and inferential statistics as well as cost estimation techniques. Our methods included both bivariate and multivariate analysis techniques to determine the socio-demographic factors that influence treatment support costs. We used one-way analysis of variance (ANOVA) to explore the socio-demographic characteristics that significantly affect treatment support costs, and a multivariate linear regression model to identify the main socio-demographic predictors of these costs. Our data met the normality assumption, as confirmed by the Shapiro–Wilk normality test. We set a significance level at a 95% confidence interval and a *P*-value of less than 0.05. We conducted our analyses using STATA version 14 and Microsoft Excel.

#### Costs estimation

The costs of supporting TB treatment include the resources required to assist a patient in treatment, as well as the value of lost productive time due to TB treatment-related activities. These costs are divided into direct and indirect costs.

Direct costs refer to the expenses incurred for transportation, food, lodging, and communication, which are paid out of pocket by the patient or their support system.

Indirect costs are the value of productive time lost to perform TB treatment support activities. The study used the human capital approach to estimate these costs, which is based on the assumption that all lost productive time should be valued in monetary equivalence [16, 17]. 

To calculate the indirect costs, treatment supporters were asked to estimate the time they spent on Directly Observed Therapy Short Course (DOTS) center visits for drug rations, waiting at the DOTS center, and observing the patient during treatment. The time was converted to days based on an average of 8 working hours per day [18], and then multiplied by the 2019 daily minimum wage rate of GH¢10.65 (US$1.99) for formal sector employees.

For informal sector employees, the study used the average daily agricultural labor wage rate of GH¢37.5 (US$7.03) in the Bono Region in November 2019. All costs were recorded in Ghana Cedis and then converted to US dollars using an average exchange rate of GH¢5.33 per US$1 (Available from:https://www.bog.gov.gh/economic-data/exchange-rate/), the interbank exchange rate in November 2019 for international comparison with other cost-of-illness studies.

### Qualitative analysis

The qualitative data were subjected to thematic analysis approaches. The gathering of data was done concurrently with analysis. The interviews' transcripts and field notes were read and reread as part of the analysis, focused on themes identified by the research team linked to the justification of coping mechanisms. These themes included borrowing of money, selling of properties, used up savings and stoppage of savings. The second author who is an expert in qualitative methods did the initial coding, which was verified by the first and fourth authors. The first and fourth authors discussed the themes in detail with the second author, who also provided indicative excerpts of the data as evidence for each theme and sub-theme. To refined the themes further, three devoted experts in the field of qualitative approaches were also given access to the codes and the preliminary results and participated in discussions in refining the findings.

The results were then presented in narrative and supported with illustrative quotes from respondents. Codes were used to identify the interviewees when quoted in the results. BHFHI-1 to BHFHI-10 means Berekum Holy Family Hospital Interviewee. SMHJSI-1 to SMHJSI-10 means Saint Mary Hospital Jaman South Interviewee. JNGHI-1 to JNGHI-10 means Jaman North Government Hospital Interviewee. DPHI-1 to DPHI-10 means Dormaa Presbyterian Hospital Interviewee.SMHI-1 to SMHI-10 means Sunyani Municipal Hospital Interviewee. TGHI-1 to TGHI-10 means Tain Government Hospital Interviewee.

#### Ethical considerations

The study was conducted in accordance with all the ethical principles involving human subject.

The Bono Regional Health Directorate as well as the various municipal and district health directorates sampled for the study were asked for their approval. All individuals provided written consent prior to participating. Participants were interviewed separately and were not obliged to reveal information that could be used to directly identify them, such as their names, in order to protect their privacy and confidentiality. The Ghana Health Service Ethics Review Committee, using the reference number GHS-ERC 003/03/2019, examined and approved the study's protocol.

## Results

### Quantitative

#### Socio-demographic characteristics of respondents

Table 3 provides a summary of the socio-demographic characteristics of the treatment supporters in this study, which had a total of 385 respondents. The mean age of the treatment supporters was 39 years (SD = 8.34). Of the respondents, 23% (90) were aged 25–34 years, 45% (174) were aged 35–44 years, and 25% (95) were aged 45–54 years. In terms of gender, 53% (205) of the treatment supporters were male, and 47% (180) were female. A total of 74% (284) were married, 17% (66) were not married, and 6% (23) were widowed.

**Table 3:** Socio-demographic characteristics of respondents (*n* = 385)

Characteristics	N	(%)
**Age group**		
18–24	13	3.4
25–34	90	23.4
35–44	174	45.2
45–54	95	24.7
> 54	13	3.4
**Gender**		
Male	205	53.3
Female	180	46.8
**Marital status**		
Never Married	66	17.1
Married	284	73.8
Widowed	23	6.0
Divorced/Separated	12	3.1
**Religion**		
Christian	303	78.7
Non-Christian	82	21.3
**Highest level of education**		
Primary	69	17.9
Junior High School	146	37.9
Senior High School	137	35.6
Tertiary (University)	33	8.6
**Ethnicity**		
Akan	77	20.0
Bono	233	60.5
Dagaaba/ Frafra/ Mo	75	19.5
**Household size**		
1	22	5.7
< 4	166	43.1
4–5	154	40.0
6–7	43	11.2
**Type of occupation**		
Farming	214	55.6
Government employee	24	6.2
Private employee	58	15.0
Trading	67	17.4
Students	6	1.6
Unemployed	16	4.2
**Sector of employment (363)**		
Formal sector	82	22.6
Informal sector	281	77.4
**Monthly income**		
No income	22	5.7
< 500	134	34.8
500–750	148	38.4
750–999	34	8.8
> 999	47	12.2
**Relationship with patient**		
Family member	276	71.7
Friend	75	19.5
Health worker	22	5.7
Spouse	12	3.1
**District of Residence**		
Berekum Municipal	68	17.7
Dormaa Municipal	54	14.0
Jaman North	48	12.5
Jaman South	38	9.9
Sunyani Municipal	104	27.0
Tain	73	19.0

NB: *Sector of Employment (363): 22 of the respondents were not economically productive*

In terms of education, 38% (146) completed junior high school, and 36% (137) completed senior high school. Of the treatment supporters, 77.4% (281) were employed in the informal sector, while 22.6% (82) were employed in the formal sector. Approximately 38% (148) earned between GH¢500- GH¢750, while 35% (134) earned less than GH¢500. On average, the monthly income was GH¢520.

Regarding their relationship with the patients, 72% (276) were family members, and 20% (75) were friends supporting the TB patients. In terms of occupation, 56% (214) were engaged in farming, 17% (67) were engaged in trading, and 6% (24) were government employees.

In terms of household size, 43.1% (166) of the treatment supporters had a household size of less than four members, while 40% (154) were living in households with a size of 4–7 persons. A total of 27% (104) of the respondents lived in Sunyani, the regional capital, 19% (73) lived in Tain, and 9.9% (38) lived in the Jaman South District.

#### Costs of TB treatment support

The monthly cost of treatment supports totaled GH¢35,837 (US$6,724). Indirect costs accounted for 67% of this amount, totaling GH¢23,853.16 (US$4,475.26), while direct costs accounted for the remaining 33%, totaling GH¢11,984.00 (US$2,248.41). The average cost of treatment support per month for the treatment supporter was GH¢112.4 (US$21.1). The primary components of the direct cost were transportation (17%) and feeding (15%). Table 4 shows the detailed results.

**Table 4:** Total cost of TB treatment support

**Cost component**	**N**	**Cost (** GH¢**)**	**Cost (**US$**)**	**Average cost (** GH¢**)**	**Average cost (**US$**)**	**Cost profile (%)**
**Direct Cost**						
Food	298	5237.0	982.6	17.8	3.3	14.6
Transportation	237	6011.0	1127.8	25.4	4.8	16.8
Others	197	736.0	138.1	3.7	0.7	2.1
**Total direct cost**		**11,984.0**	**2248.4**	**46.8**	**8.7**	**33.4**
**Indirect Cost**						
Formal Sector	82	1530.9	287.2	18.7	3.5	4.3
Informal Sector	281	22,322.2	4188.0	79.4	14.9	62.3
**Total Indirect Cost**	**363**	**23,853.2**	**4475.3**	**65.7**	**12.3**	**66.6**
**TOTAL COST**		**35,837.2**	**6723.7**	**112.4**	**21.1**	**100**

Please note that the national minimum wage for 2019 in Ghana was GH¢10.7, which was used to calculate the value of productivity hours lost by formal sector supporters. For informal sector treatment supporters, the local agricultural wage rate of GH¢37.5 was used. As of November 2019, the exchange rate was GH¢5.33 to US$1

#### Bivariate analysis: mean difference of TB treatment support costs by socio-demographic characteristics of respondents

According to Table 5, the one-way ANOVA test was used to assess the mean direct, indirect, and total costs of TB treatment support based on the socio-demographic characteristics of the study participants. The results indicated significant differences in the mean TB treatment support cost among different groups. For instance, the total direct cost of TB treatment support varied significantly based on religion (T = -2.2, *p* = 0.03), highest level of education (F = 9.0, *p* < 0.001), household size (F = 10.2, *p* < 0.001), monthly income (F = 5.7, *p* < 0.001), and district of residence (F = 6.7, *p* < 0.001).

**Table 5:** Bivariate analysis: mean differences of TB treatment support costs by socio-demographic characteristics of respondents

Characteristics	Total direct cost				Total indirect cost			Total cost		
	**Mean**	**SD**	**F-stat; *P*-value**		**Mean**	**SD**	**F-stat; *P*-value**	**Mean**	**SD**	**F-stat; *P*-value**
**Age group**				0.8; 0.562			1.0; 0.434			1.0; 0.400
18–24	34.6	16.4			71.5	30		106.2	39.1	
25–34	31.7	11.5			56.4	33		88.1	33.9	
35–44	30.1	12			61.9	29.7		92.1	31.2	
45–54	32	12.2			61	30.2		93.1	31.4	
> 54	30.8	14			64.2	34.3		95	32.7	
**Gender**				-1.1 T; 0.254			-6.9 T; < 0.001***			-7.1 T; < 0.001***
Male	30.5	11.9			51.3	28.7		81.7	30.9	
Female	31.9	12.4			71.7	29.4		103.6	29.7	
**Marital status**				0.4; 0.772			3.8; 0.010 *			2.6; 0.050 *
Never Married	31.7	12.5			56.8	41.1		88.5	35.8	
Married	31.1	12.1			60.7	29.3		91.8	32.7	
Widowed	31.2	12			59.7	0		90.9	12	
Divorce	27.7	13.2			88.9	0		116.6	13.2	
**Religion**				2.2 T; 0.030 *			-8.4 T; < 0.001***			-6.6 T; < 0.001***
Christian	31.8	12.4			56	31.8		87.8	33.6	
Non-Chris	28.7	10.8			78.5	17.8		107.3	20.1	
**Highest education**				9.0; < 0.001***			37.5; < 0.001***			21.3; < 0.001***
Primary	29.4	10.7			70.8	11		100.2	14.1	
Junior high	29.8	12.5			73.5	28.8		103.3	33.6	
Senior high	31	11.2			50.4	27.6		81.5	28.6	
Tertiary	41.1	13.1			27	38.8		68.1	40.7	
**Ethnicity**				2.4; 0.091			7.9; < 0.001***			7.1; 0.001 **
Akan	33.7	13.1			56.2	35.4		89.9	33	
Bono	30.2	11.9			58.4	29.8		88.7	32.4	
Dagaaba/ Frafra/ Mo	31.2	11.7			73.1	25.4		104.3	27.9	
**Household size**				10.2; < 0.001***			40.0; < 0.001***			22.6; < 0.001***
1	41.3	12			0	0		41.3	12	
< 4	29.2	10.5			65.1	26.9		94.4	29.6	
4–5	30.2	11.5			64.8	27.8		95	30.1	
6–7	36.4	16			61.2	30.1		97.7	35.3	
**Employment sector**				-0.4 T; 0.687			-33.2^**T**^; < 0.001***			-25.2^**T**^; < 0.001***
Formal	30.7	10.9			18.5	7.9		49.2	14.5	
Informal	31.2	12.5			72.3	23.8		103.5	25.2	
**Monthly income**				5.7; < 0.001***			53.4; < 0.001***			34.4; < 0.001***
No income	41.3	12			0	0		41.3	12	
< 500	32.2	12.5			71.3	26.5		103.5	29.8	
500–750	28.9	11.9			65.1	26.9		94	30	
750–999	30.1	8.1			73.3	7.6		103.4	10.4	
> 999	31.1	12			37	25.9		68.1	27.9	
**Relationship to patient**				0.4; 0.745			29.1; < 0.001***			24.8; < 0.001***
Family member	31.5	12.5			60.8	30.7		92.3	32.4	
Friend	29.7	11.7			75	22.3		104.7	23.7	
Health worker	31	9.8			12	0		43	9.8	
Spouse	31.3	10			63.2	0		94.5	10	
**District**				6.7; < 0.001***			0.1; 0.985			1.2; 0.296
Berekum	30	12.7			59.8	31.1		89.7	33.5	
Dormaa	35.8	13.3			63.3	30.6		99.1	31.6	
Jaman North	35.5	14.5			59	32		94.5	32.8	
Jaman South	25.5	6.2			59.8	29.2		85.3	28.7	
Sunyani	28.2	8.6			61	30.9		89.2	31.2	
Tain	33.1	13.5			61.5	31.2		94.5	33.9	

*SD* standard deviation. *: *p* < 0.05. **: *p* < 0.01. ***: *p* < 0.001. T: Welch’s t-test value

Similarly, significant variations were observed in the mean total indirect cost of TB treatment support based on the sex of respondents (F = 47.4, *p* < 0.001), marital status (F = 3.8, *p* = 0.01), religion (T = -8.4, *p* < 0.001), highest level of education (F = 37.5, *p* < 0.001), ethnicity (F = 7.9, *p* < 0.001), household size (F = 40.0, *p* < 0.001), employment sector (F = 404, *p* < 0.001), monthly income (F = 53.4, *p* < 0.001), and relationship with the patient (F = 29.1, *p* < 0.001).

Lastly, the total mean cost of TB treatment support varied significantly based on the sex of respondents (F = 49.7, *p* < 0.001), marital status (F = 2.6, *p* = 0.05), religion (T = -6.6, *p* < 0.001), highest level of education (F = 21.3, *p* < 0.001), ethnicity (F = 7.1, *p* = 0.001), household size (F = 22.6, *p* < 0.001), employment sector (F = 350.2, *p* < 0.001), monthly income (F = 34.4, *p* < 0.001), and relationship with the patient (F = 24.8, *p* < 0.001).

The study utilized a multivariate joint linear regression model to analyze the factors affecting the costs of TB treatment among participants. The findings revealed notable differences in the total cost of treatment support based on marital status, religion, education level, household size, and district of residence.

Widows had an average total direct cost of GH¢26.0 (95% CI: GH¢ [7.1, 44.8]), which was significantly higher than those who had never been married. Participants with tertiary education had an average total direct cost of GH¢12.1 (95% CI: GH¢ [5.2, 19.0]), which was significantly higher than those with primary education.

Moreover, an increase in the number of household members resulted in a GH¢1.1 (95% CI: GH¢ [0.2, 2.0]) rise in total direct cost. The area of residence also played a role, with the average total direct cost of TB treatment being significantly higher in Dormaa and Jaman North districts than in Berekum District. Conversely, the Jaman South District had a significantly lower average total direct cost of GH¢4.7 (95% CI: GHS [-9.2, -0.2]).

The joint effect results of the multivariate linear regression demonstrated that marital status, religion, education level, household size, monthly income, and district of residence significantly influenced the direct, indirect, and total cost of TB treatment support among participants (F = 3.0, *p* = 0.029; F = 10.3, *p* = 0.002; F = 4.7, *p* = 0.003; F = 6.2, *p* = 0.002; F = 28.7, *p* < 0.001; F = 7.2, *p* < 0.001). Please refer to Table 6 for more details.

**Table 6:** Multivariate linear regression model: association between socio-demographic characteristics and cost of TB treatment support

	**Total direct cost**	**Total indirect cost**	**Total cost**	**Joint effect**
**Characteristics**	**aβ [95% CI]**	**aβ [95% CI]**	**aβ [95% CI]**	**F-stat; *P*-value**
**Age**	-0.1 [-0.2, 0.1]	0.0 [-0.1, 0.2]	0.0 [-0.3, 0.2]	0.4; 0.641
**Gender**				2.7; 0.100
Male	0.0 (reference)	0.0 (reference)	0.0 (reference)	
Female	4.0 [-0.8, 8.8]	-27.1 [-32.4, -21.8] ***	-23.1 [-30.7, -15.5] ***	
**Marital status**				
Never married	0.0 (reference)	0.0 (reference)	0.0 (reference)	3.0; 0.029 *
Married	2.8 [-2.9, 8.6]	29.2 [22.9, 35.6] ***	32.1 [23.0, 41.2] ***	
Widowed	26.0 [7.1, 44.8] **	-42.0 [-62.9, -21.0] ***	-16.0 [-46.0, 14.0]	
Divorce	-2.1 [-11.6, 7.3]	38.4 [27.9, 48.9] ***	36.2 [21.2, 51.3] ***	
**Religion**				
Christian	0.0 (reference)	0.0 (reference)	0.0 (reference)	10.3; 0.002 **
Non-Chris	-7.4 [-12.0, -2.9] **	19.2 [14.1, 24.2] ***	11.7 [4.5, 19.0] **	
**Highest education**				4.7; 0.003 **
Primary	0.0 (reference)	0.0 (reference)	0.0 (reference)	
Junior high	13.1 [-4.8, 30.9]	-8.2 [-28.1, 11.6]	4.8 [-23.5, 33.2]	
Senior high	15.2 [-2.8, 33.1]	-19.6 [-39.6, 0.3]	-4.4 [-33.0, 24.1]	
Tertiary	12.1 [5.2, 19.0] **	-48.6 [-56.3, -40.9] ***	-36.5 [-47.5, -25.6] ***	
**Ethnicity**				2.9; 0.057
Akan	0.0 (reference)	0.0 (reference)	0.0 (reference)	
Bono	-15.4 [-32.2, 1.4]	9.3 [-9.3, 28.0]	-6.1 [-32.7, 20.6]	
Dagaaba/ Frafra/ Mo	-11.7 [-28.1, 4.6]	9.9 [-8.3, 28.0]	-1.9 [-27.9, 24.1]	
**Household size**	1.1 [0.2, 2.0] *	1.4 [0.4, 2.4] **	2.5 [1.1, 3.9] ***	6.2; 0.002 **
**Employment sector**				0.1; 0.722
Formal	0.0 (reference)	0.0 (reference)	0.0 (reference)	
Informal	-0.9 [-5.9, 4.1]	75.5 [70.0, 81.0] ***	74.6 [66.8, 82.5] ***	
**Monthly income (₵100)**	0.0 [-1.0, 0.9]	-4.1 [-5.2, -3.0] ***	-4.2 [-5.7, -2.6] ***	28.7; < 0.001***
**Relationship to patient**				0.3; 0.840
Family member	0.0 (reference)	0.0 (reference)	0.0 (reference)	
Friend	-0.7 [-4.1, 2.7]	2.4 [-1.3, 6.2]	1.7 [-3.7, 7.1]	
Health worker	-0.4 [-8.7, 7.9]	21.6 [12.3, 30.8] ***	21.2 [7.9, 34.4] **	
Spouse	-4.0 [-14.1, 6.1]	-0.4 [-11.6, 10.8]	-4.4 [-20.4, 11.6]	
**District**				7.2; < 0.001***
Berekum	0.0 (reference)	0.0 (reference)	0.0 (reference)	
Dormaa	6.3 [2.3, 10.4] **	-1.2 [-5.7, 3.3]	5.1 [-1.3, 11.6]	
Jaman North	4.8 [0.5, 9.1] *	-1.9 [-6.7, 2.8]	2.9 [-3.9, 9.7]	
Jaman South	-4.7 [-9.2, -0.2] *	-4.7 [-9.7, 0.3]	-9.4 [-16.6, -2.2] *	
Sunyani	-1.8 [-5.2, 1.7]	-0.3 [-4.1, 3.6]	-2.0 [-7.5, 3.5]	
Tain	3.1 [-0.6, 6.9]	-0.8 [-5.0, 3.3]	2.3 [-3.6, 8.3]	

*aβ* adjusted coefficient, *CI* confidence interval

^*^p < 0.05

^**^p < 0.01

^***^p < 0.001

### Qualitative result

#### Demographic characteristics of the Interviewees

Of the 60 individuals interviewed in depth, 39 (65%) were male and 21 (35%) were female. Their average age ranged from 30 to 45 years old. Of those interviewed, 47 (78.3%) were married, while 13 (21.7%) were not. The majority of those interviewed, 53 (88.3%), work in agriculture. In regards to education, 41 had attained a senior high school education, while 19 had obtained a junior high school education.

#### Coping mechanisms by treatment supporters

During a qualitative study on the experiences of TB treatment supporters and their coping mechanisms, interviewees revealed that this support work was voluntary and that supporters had to bear all the direct and indirect costs themselves. This was especially difficult in a country without a well-established social welfare benefit system or provisions for the unemployed or disabled. To cope with the financial burden of supporting TB treatment, supporters described various coping mechanisms. The findings are presented in four sub-themes: selling properties, using savings, stopping savings, and borrowing money.

#### Borrowing of money

After analyzing the data, it was found that unexpected expenses and additional support tasks caused financial instability for most of the caregivers. In some cases, caregivers had to sacrifice work hours, which resulted in a loss of income. To manage the extra costs, caregivers often borrowed money from their support network, including friends and family.

*…I owe people some money; I borrowed some money from a family member, he gave me* GH¢*25.00 and one of my friends also gave me* GH¢*20.00… Altogether, it is* GH¢*45.00… Still, I haven’t been able to pay anyone of them…” (BHFHI-3).*

*“…Since I started supporting my husband with his TB treatment, no one has ever offered us any financial help, I have to borrow money from other family members and pay back whenever I sell my farm produce…” (TGHI-6).*

*“…Because I borrow to support my wife tuberculosis treatment that have left me with a lot of unsettled debt, last month for instance, I couldn’t pay for my light and water bills…” (SMHI-7).*

### Use of personal savings

Some of the treatment supporters had used up their personal savings that had been allocated for life’s fulfilling activities, such as paying children’s school fees, building a house, funeral, among others:

*“…I was saving for my children’s school fees, but used that money to pay for transportation to the hospital for TB drugs for the treatment of my son who has tuberculosis…” (SMHI-5).*

*“…now that I am a treatment supporter, I have to spend the little money that I have been able to save on my friend’s treatment to see how he can get well…the transportation cost and time spent traveling to the hospital is a problem for me…” (TGHI-4).*

Treatment supporters used their own savings to help cover costs of tuberculosis treatment.

### Sale of personal properties

Treatment supporters often face costs related to treatment that are not reimbursed, which can create financial strain. To make ends meet, some supporters have resorted to selling their assets, such as clothing, farm produce, or livestock. This can have severe consequences for their socio-economic status and future well-being. Supporters describe the difficult decisions they must make in order to support TB patients undergoing treatment.

*“…I sold my clothes in order to support my husband with the treatment of his tuberculosis… I have to fall on anything around to raise money for my husband’s treatment…*” *(SMHJSI-7).*

*“…The most important thing to me as a treatment supporter is to see my friend free from tuberculosis…. the other day I had to sell my reserve tubers of yams to raise some money for my friend’s tuberculosis treatment” … (DPHI-2).*

Selling personal property to pay for healthcare expenses can deplete resources and increase the risk of impoverishment.

### Stoppage of savings

Treatment supporters face challenges in saving money for other activities due to loss of income and increasing financial demands while providing treatment support. They express frustration at not being able to save money while supporting TB patients with treatment.

*“…I could put some money down for the future when I was not taking care of my friend with the TB treatment, now I have to spend the little money that I have on my friend’s treatment to see how s/he can get well… the transportation cost and time spent traveling to the hospital is a problem for me…” (BHFHI-1).*

*“…I spent all my money in helping out with the transportation costs since I started supporting my mother with treatment without any financial assistance from anywhere…. this makes it difficult for me to save any money for now…” (JNGHI-10)*.

The cost of treatment support can prevent supporters from saving, impacting their socio-economic wellbeing and future welfare.

## Discussion

The study revealed that almost half (45%) of the participants were between the ages of 35 and 44, with the majority (72%) being family members. This indicates that those who are employed have to take time off work to provide unpaid support for TB treatment. This can affect not only the supporter's income but also the entire family's financial situation, especially if the supporter is the main breadwinner. The study emphasizes the importance of family support during times of need, as seen in Pakistan where most TB treatment supporters were family members and community volunteers [5]. Similar findings were also reported in a study on caregivers of patients with mental illness in Ghana, where is was found that most caregiver were family members [20].

According to our study, the average monthly cost of providing treatment to a tuberculosis patient was GH¢112.4 (US$21.1) and more than half (50.2%) of the direct expenses were due to transportation costs for obtaining medication. Similar studies have found that transportation expenses are the largest component of direct costs for TB treatment due to the frequency, and mode of transportation required to reach the hospital [21, 22]. The study's findings align with previous research conducted in Zambia, which found that caregivers incurred greater transportation expenses than other cost components [23].

The study found that several factors, including religion, education level, household size, monthly income, and district of residence, were identified as the predictors of the direct cost of providing TB treatment support. These findings are consistent with previous studies conducted in Ethiopia, Benin, and China that found education, income, and place of residence to be significant factors in TB treatment costs [10, 18, 24].. Another study conducted in Ethiopia found that gender, education level, and rural residency were also significant factors [25].

Overall, the study highlights the various direct costs associated with providing TB treatment support and the need to consider factors such as education, income, and place of residence when addressing these costs.

When estimating indirect costs, we discovered that the size of a household can impact the total indirect cost of supporting tuberculosis patients during treatment. This means that treatment supporters from larger households are more likely to experience indirect costs than those from smaller households. This aligns with a previous study that identified larger households as an independent predictor of the total indirect cost associated with illness care [6].

Additionally, we found that treatment supporters with a higher level of education are more likely to incur lower costs compared to those with a lower level of education. This finding is consistent with previous studies, such as one conducted in Nigeria where researchers found that the level of education attainment significantly influenced the indirect costs incurred by households [26]. Another study in Benin also found that educational attainment was an independent predictor of the indirect costs associated with seeking tuberculosis treatment [10].

In-depth interviews revealed that the costs associated with supporting tuberculosis treatment were significant for the treatment supporters. They had to spend money on food and transportation, and also lose productive time for visits to health facilities for drug ration. To cope with these costs, treatment supporters resorted to borrowing money from their support network, selling assets, using up personal savings, or stopping savings altogether. Our study compared these findings with those of previous research on coping mechanisms.

It appears that rural patients face lower availability and quality of care, but higher absolute and relative costs due to the need to travel and lower incomes, respectively [23, 27]. They are more likely to resort to coping mechanisms, especially since socioeconomic advantages, such as access to jobs and educational opportunities, quality of roads, and availability of frequent public transport, are limited [28].

Our findings are consistent with previous work suggesting that TB patients and their households cope through the sale of assets and borrowing of money from their support network [29]. Therefore, we suggest paying more attention to the financial burden associated with TB treatment support and the strategies that treatment supporters use to navigate this burden. Providing varied financial support and targeted investment for treatment supporters, which takes into account the cost of transport and loss of work, will be essential to financially protect individuals who are assisting TB patients with treatment and improve health outcomes. This would ensure better outcomes for cost mitigation and reduction of financial barriers for access to TB treatment, as well as treatment adherence and monitoring of progress towards the End TB strategy target of zero TB-associated costs for all parties [28, 29]. Additionally, further qualitative exploration of the psycho-social impacts of assisting TB patients with treatment would strengthen the development and implementation of any interventions.

However, in the context of our study, it is important to acknowledge that the data and findings presented here are based on research conducted in 2019. Since that time, many factors and circumstances may have evolved, which could potentially impact the relevance and applicability of our results to the current situation.

### Strength and limitation of the study

This study differed from previous ones by estimating both the costs and coping mechanisms associated with TB treatment support, rather than just the costs to patients, households, and the health system. However, it only included treatment supporters who were assisting patients registered with DOTS centres, which limits the representativeness of the cost estimates to the entire population of individuals supporting TB patients. Additionally, challenges with accurately recalling treatment-related expenditures may have led to underestimation of costs due to recall bias. Therefore, researchers and policymakers should exercise caution when using these estimates, as they may not accurately represent the true costs incurred by treatment supporters.

## Conclusion

The estimated costs and coping mechanisms associated with supporting tuberculosis patients with treatment are significant and may worsen the socio-economic status and future welfare of treatment supporters. These costs are incurred by respondents to ensure access to treatment, prevent defaulting, and enhance completion of the treatment.

Considering our findings, we recommend that policies should be directed towards reducing these costs as a step towards achieving the End TB goals.

### Supplementary Information

**Additional file 1. **Interview Guide for Tuberculosis Treatment Supporters in Bono Region, Ghana.

## Data Availability

The datasets generated and/or analyzed during the current study are not publicly available due to concern for participants privacy because of the silent stigma associated with tuberculosis and also the data was collected among treatment supporters registered in government hospitals, hence, sharing the data may have legal implications. But are available from the corresponding author bagngmen@gmail.com on reasonable request.
