# Supplementary material for: Economic burden and coping mechanisms by tuberculosis treatment supporters: a mixed method approach from Bono Region, Ghana
Source: BMC Health Serv Res. 2024 Jan 30;24:148. doi: 10.1186/s12913-024-10611-1 (PMC10826066; doi:10.1186/s12913-024-10611-1)
Supplement: Supplementary file 1 — Additional file 1. Interview Guide for Tuberculosis Treatment Supporters in Bono Region, Ghana. [file 12913_2024_10611_MOESM1_ESM.docx]

**Interview Guide for Tuberculosis Treatment Supporters in Bono Region, Ghana**

**Study Title:** Economic Burden of Tuberculosis to Treatment Supporters in Bono Region, Ghana

**Part A: Consent Form**

**Participants’ Statement**

I acknowledge that I have read or have had the purpose and contents of the Participants’ Information Sheet read and all questions satisfactorily explained to me in a language I understand (English [ ] /Twi [ ] /Bono[ ]). I fully understand the contents and any potential implications as well as my right to change my mind (ie withdraw from the research) even after I have signed this form.

I voluntarily agree to be part of this research.

Name of Participant…………………………..

Participants’ Signature/Thumb Print………… Date………………………………….

**Permission for Audio Tape Recording**

Do you voluntarily agreed to be recorded?

Yes [ ] No [ ]

**Interpreters’ Statement**

I interpreted the purpose and contents of the Participants’ Information Sheet to the afore named participant to the best of my ability in the (Bono [ ] / Twi [ ]) language to his proper understanding. All questions, appropriate clarifications sort by the participant and answers were also duly interpreted to his/her satisfaction.

Name of Interpreter……………………………

Signature of Interpreter……………………….. Date…………………….

**Statement of Witness**

I was present when the purpose and contents of the Participant Information Sheet was read and explained satisfactorily to the participant in the language he/she understood (English [ ] /Twi [ ] / Bono [ ]).

I confirm that he/she was given the opportunity to ask questions/seek clarifications and same were duly answered to his/her satisfaction before voluntarily agreeing to be part of the research.

Name……………………………….

Signature/Thumb Print ………................ Date………………………….

**Investigator Statement and Signature**

I certify that the participant has been given ample time to read and learn about the study. All questions and clarifications raised by the participant have been addressed.

Researcher’s name……………………………………….

Signature ……………………… Date…………………………………

**Parts B: In-Depth Interview Guide for Treatment Supporters**

Study Title: economic burden of tuberculosis treatment supporters in Bono Region, Ghana

**Date of interview**___________________________

**Name of Interviewer**_________________________

**Demographic Characteristics**

1. Age
2. Sex
3. Marital status
4. Occupation
5. Level of education

This interview will take approximately (30-45) minutes. First, I will ask you some very specific questions about the possible financial impact of providing DOTS support. Then we’ll move to more general questions and conversation about the overall significance of these extra costs in your life.

**Coping Mechanisms**

1. How long have you been a treatment supporter?
2. How many patients have you supported since you started?
3. Tell me about your experiences of being a treatment supporter?
4. Have you incurred costs due to being a treatment supported? That’s expenses you would not have incurred if you had not being a treatment supporter?
5. Tell me about the major expenses that you’ve noticed since you started working as DOTS supporter? (Probes: money spent on meals, or food, accommodations, telephone calls, transportation and others).
6. Briefly, can you give me a sense of how you have managed/been coping with these extra expenses?
7. I’m going to list a number of things that might have happened as a result of these extra costs/expenses. Please tell me if any of them have happened to you:

Probes

1. Gone into debt or increased your debt
2. Used up savings?
3. Stopped saving?
4. Sold property or other assets
5. Borrowed money from support networks (e.g, family, friends etc)
6. Remove children from school/change school to less expensive one?
7. Skipped meals?
8. Restricted what your children buy?
9. How has your job/employment been affected?

Probes:

- Reduced hours at work
- Reduce income
- Absenteeism from work

1. Finally, is there anything that we have not talked about that you think would be important for us to know?

**Thank you very much for your time and input.**
